# A rare presentation of anaplastic large cell lymphoma as a cavitary pulmonary mass with hypercalcemia

**DOI:** 10.1111/1759-7714.14571

**Published:** 2022-07-10

**Authors:** Hsu‐Yuan Chen, Yu‐Chu Kuo, Wen‐Chien Cheng, Wei‐Cheng Chen

**Affiliations:** ^1^ Department of Internal Medicine China Medical University Hospital Taichung Taiwan; ^2^ Division of Pulmonary and Critical Care Medicine, Department of Internal Medicine China Medical University Hospital Taichung Taiwan

**Keywords:** anaplastic large cell lymphoma, cavitary pulmonary mass, hypercalcemia, lung cavity, lymphoma

## Abstract

Cavitary lung lesions found on chest imaging may point to various diseases. These lesions may be caused by numerous etiologies, such as infection, inflammatory diseases, or malignancy. Thus, its etiology may be challenging to differentiate using imaging alone. Differential diagnoses are created using a combination of clinical symptoms, medical history, laboratory results, and physical examination. Primary pulmonary lymphoma and anaplastic large cell lymphoma (ALCL) are rare differentials. Here, we report a case of ALCL that initially presented with back pain, intermittent fever, hemoptysis, hypercalcemia, and bilateral multiple cavitary lung nodules. Because a cavitary pulmonary mass with sustained hypercalcemia is commonly seen in patients with squamous cell carcinomas or pulmonary tuberculosis, patients with ALCL may be misdiagnosed and undergo delayed treatment. This study highlights that ALCL should be considered in patients presenting with a cavitary pulmonary mass and hypercalcemia.

## Graphical abstract

Various diseases may present as cavitary pulmonary nodules. This case highlights that anaplastic large cell lymphoma should be considered in patients presenting with cavitary masses and sustained hypercalcemia.

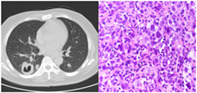

## INTRODUCTION

Lung cavitary disease involves various pathological processes, making its diagnosis challenging. Its differential diagnoses can be narrowed down by a thorough history taking and including other vital clinical evidence. Squamous cell carcinoma (SqCC) commonly presents with cavitary pulmonary masses and hypercalcemia. Other possible differential diagnoses of pulmonary masses with hypercalcemia include tuberculosis, sarcoidosis and chronic fungal infections. Although uncommon, pulmonary masses with hypercalcemia may also occur in patients with anaplastic large cell lymphoma (ALCL). This disease has a pathological morphology similar to that of carcinomas. Lung involvement occurs in 11% of ALCL cases, with radiographic features that include nodules, masses, airspace consolidation and cavitation.^1^ Case reports on ALCL with cavitary pulmonary lesions remain limited.^1^, ^2^, ^3^ To our knowledge, this is the first study to present a case of ALCL that presented with a cavitary pulmonary mass and hypercalcemia.

## CASE REPORT

**FIGURE 1:**
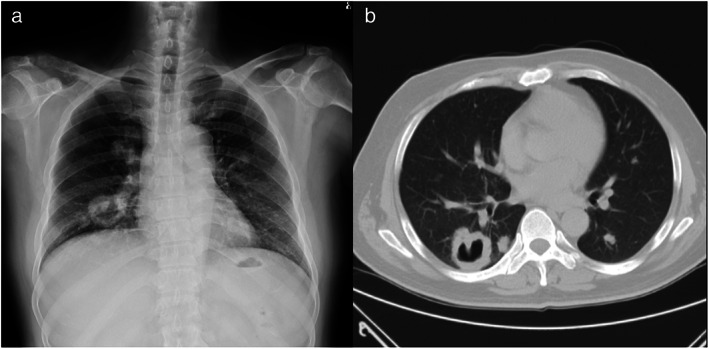
(a) Chest radiograph revealed a well‐defined mass, with a cavity over the right lower lung field. Multiple nodules were noted in the other lung fields. (b) Computed tomography revealed a well‐defined, thick‐walled cavitary mass with an irregular inner border. Two other nodules were identified

A 48‐year‐old hypertensive male smoker presented with a two‐week history of intermittent fever reaching up to 39°C, accompanied by severe back pain, weight loss of 20 kg in the past 6 months, and coughing with purulent blood‐tinged sputum. Chest radiography revealed a well‐defined cavitated mass over the right lower lung field, multiple scattered pulmonary nodules, and osteolytic lesions in the right clavicle (Figure 1a). Computed tomography revealed a well‐defined, thick‐walled cavitary mass with irregular inner borders, several pulmonary nodules, and multiple osteolytic lesions (Figure 1b).

**FIGURE 2:**
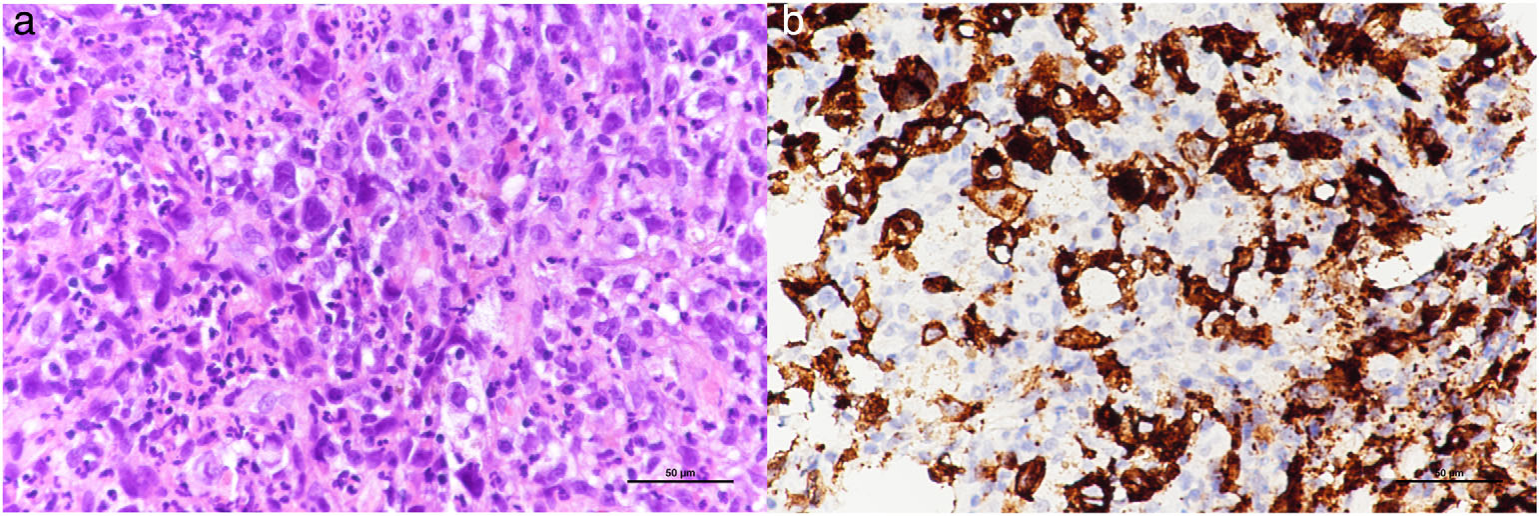
(a) Hematoxylin–eosin staining revealed isolated large atypical lymphocytes with abundant cytoplasm (b) Immunohistochemical staining showed infiltration of CD30‐positive large anaplastic cells (CD30 immunostaining, 400×)

On physical examination, he was disoriented, febrile (39.4°C), hypertensive (136/97 mmHg), and tachycardic (133 beats/min). He had a respiratory rate of 18 breaths/min, and an oxygen saturation level of 100% while on a 3 L/min nasal cannula. Bilateral rales were noted on auscultation. Laboratory examination revealed an elevated leukocyte count (28 100/ml) with a differential of 92% neutrophils, 2.7% lymphocytes, and 4.1% monocytes, elevated C‐reactive protein level (17.6 mg/dl), decreased hemoglobin (8.5 g/l), a platelet count of 274 000/ml, elevated serum creatinine levels (1.62 mg/dl), elevated corrected serum calcium levels (13.5 mg/dl) and elevated lactate dehydrogenase (346 IU/l). Since an infectious disease or malignancy was highly likely, ceftaroline and voriconazole were given as empiric therapy. Hydration and zoledronic acid were administered to manage his hypercalcemia. His sputum yielded negative results for serum *Mycoplasma pneumoniae* immunoglobulin M serum and *Aspergillus galactomannan*. An endobronchial ultrasound‐guided biopsy of the pulmonary mass was performed. The presence of isolated large atypical lymphocytes with abundant cytoplasm on pathological examination led to the diagnosis of ALCL. Immunohistochemical staining was positive for common leukocyte antigen and CD30, while negative for CD3, CD20, CD79a, TTF‐1, p40, p53, CK (AE1/AE3), EBER, Alk, and CD15 (Figure 2a,b).

However, the patient underwent pulseless ventricular tachycardia in the ward, suspectedly due to electrolyte disturbances. Spontaneous circulation returned after 8 min of cardiopulmonary resuscitation and defibrillation. Although targeted temperature management and anticonvulsive medications were initiated, the patient was still in a deep coma due to hypoxic encephalopathy‐related status epilepticus. No further lymphoma‐related therapy is administered to comatose patients who are dependent on mechanical ventilation.

## DISCUSSION

ALCL uncommonly presents as a pulmonary mass on imaging. Moreover, it rarely presents as a cavity in a patient with hypercalcemia. Various etiologies lead to pulmonary cavitary diseases, and its diagnosis may be narrowed down through a thorough history taking and other clinical parameters. Cavities with a maximum wall thickness > 15 mm, irregular internal walls, absence of centrilobular nodules, and upper lobe predominance point to a malignant etiology.^4^, ^5^, ^6^, ^7^ Our patient was a smoker who presented with a pulmonary cavitary mass and hypercapnia, pointing towards SqCC as a likely cause.^8^ Other possible differential diagnoses for a pulmonary mass in a patient with hypercalcemia include tuberculosis, sarcoidosis, chronic fungal infections, and lymphomas.^9^ Hypercalcemia is caused by osteoclastic bone resorption, humoral hypercalcemia, 1,25(OH)2D3‐secreting lymphoma, and ectopic hyperparathyroidism.^10^ Hypercalcemia is less frequently seen in patients with ALCL. Only one previous case report mentioned 1,25(OH)2D3‐mediated hypercalcemia in a patient with ALCL.^11^ In our patient, his hypercalcemia was likely due to his multiple osteolytic lesions. Establishing the etiology of pulmonary cavities through noninvasive testing may be challenging. This is further made difficult due to its nonspecific clinical symptoms and radiographic features. Invasive diagnostic methods, such as bronchoscopy, may be utilized. To our knowledge, this is the first study to present a case of ALCL that presented with a cavitary pulmonary mass and hypercalcemia.

ALCL is a newly described type of non‐Hodgkin lymphoma, with a typical T cell phenotype. This disease has an aggressive clinical course. It is characterized by pleomorphic large cells expressing the CD30 antigen.^1^, ^3^ Primary systemic ALCL accounts for 8% of non‐Hodgkin lymphomas cases.^12^ Although lung involvement may occur as a result of dissemination, primary pulmonary ALCL is rarely reported.^2^ While ALK‐positive patients have a superior prognosis than those who are ALK‐negative (five‐year overall survival, 70% vs. 49%; *p* = 0.016),^13^ our ALK‐negative patient had an approximately 50% five‐year survival rate if the diagnosis of ALCL had been made earlier.

Various diseases may present as cavitary pulmonary nodules. This case highlights that ALCL should be considered in patients presenting with cavitary masses and sustained hypercalcemia.

## CONFLICT OF INTEREST

The authors declare that they have no conflict of interest.
